# Supplementary material for: Complexome profiling of the Chlamydomonas psb28 mutant reveals TEF5 as an early PSII assembly factor
Source: Plant Cell. 2025 Mar 17;37(6):koaf055. doi: 10.1093/plcell/koaf055 (PMC12164590; doi:10.1093/plcell/koaf055)
Supplement: koaf055_Supplementary_Data [file koaf055_supplementary_data.zip › psb28_interactive.zip!/plots/intensity/qualityPlots/peptides/_Cre12.g514200.t1.2.html]

### Cre12.g514200.t1.2

Trivial name:   
SeqCoverage [median]: 44.80  
Mass: 64.46 kDa  
  
 

| Sequence | MissedCleavage | Mass [Da] | wtIntensity | muIntensity | Score |
| --- | --- | --- | --- | --- | --- |
| AAALLRGQATIAPSRQPVAV | 2 | 1989.15 | 1314.20 | 0.00 | 95.95 |
| ASSDKDLEEYIR | 1 | 1424.68 | 7346.52 | 1057.09 | 87.00 |
| ASSDKDLEEYIRR | 2 | 1580.78 | 11990.79 | 0.00 | 84.72 |
| EHGIDVVSDLSGVGQNLQDHPAAVLAAR | 0 | 2867.45 | 15318.46 | 6183.41 | 133.95 |
| EIYLAR | 0 | 763.42 | 4001.94 | 0.00 | 126.25 |
| GQATIAPSR | 0 | 899.48 | 3731.12 | 563.10 | 158.61 |
| GQATIAPSRQPVAV | 1 | 1393.77 | 4389.65 | 675.11 | 124.42 |
| IPGGQTGAATVMVAER | 0 | 1556.80 | 10037.17 | 2394.35 | 135.14 |
| KIAAQEPLKK | 2 | 1124.69 | 9467.05 | 7096.46 | 152.07 |
| LFAHPVMDWGMSSLTQK | 0 | 1946.94 | 5393.20 | 0.00 | 104.95 |
| LGAVAQYLFQR | 0 | 1264.69 | 5487.10 | 0.00 | 184.49 |
| MLGGSSGSNATLYHR | 0 | 1549.73 | 7795.55 | 2178.34 | 73.02 |
| SYIVFGELK | 0 | 1054.57 | 914.60 | 0.00 | 63.48 |
| SYIVFGELKK | 1 | 1182.66 | 8547.11 | 1094.25 | 67.73 |
| TSSSLSQPDLQMR | 0 | 1448.69 | 12672.56 | 4720.70 | 206.27 |
| TTGVEYAMQQFGDR | 0 | 1601.71 | 7722.96 | 352.29 | 142.97 |
| TVHSGNALVGTAAMGASPAAGAVVSSADLK | 0 | 2709.38 | 11411.61 | 7370.31 | 88.84 |
| TYLKPAMAR | 1 | 1049.57 | 14468.69 | 3475.47 | 73.98 |
| VFGVEGLR | 0 | 875.49 | 20818.67 | 8325.77 | 158.12 |
| VLVLEAGPTGDAMEVAVPAGITR | 0 | 2265.20 | 25023.26 | 6306.88 | 126.02 |
| VVDASVLPR | 0 | 954.55 | 29823.69 | 10791.69 | 146.58 |
| YENVLHDEFFK | 0 | 1439.67 | 11601.91 | 14243.22 | 114.24 |
| YLQEETFPGER | 0 | 1367.64 | 17910.85 | 8374.94 | 177.57 |
|  |  |  | 247188.7 | 85203.4 |  |
